# Thymol Increases Sensitivity of Clinical Col-R Gram-Negative Bacteria to Colistin

**DOI:** 10.1128/spectrum.00184-22

**Published:** 2022-06-14

**Authors:** Zhuocheng Yao, Luozhu Feng, Yining Zhao, Xiaodong Zhang, Lijiang Chen, Lingbo Wang, Ying Zhang, Yao Sun, Tieli Zhou, Jianming Cao

**Affiliations:** a Department of Clinical Laboratory, The First Affiliated Hospital of Wenzhou Medical University; Key Laboratory of Clinical Laboratory Diagnosis and Translational Research of Zhejiang Province, Wenzhou, Zhejiang Province, China; b Department of Medical Lab Science, School of Laboratory Medicine and Life Science, Wenzhou Medical University, Wenzhou, Zhejiang Province, China; Northwestern University

**Keywords:** Gram-negative bacteria, colistin-resistant, thymol, biofilm, synergy effect, microbial infection, bacterial resistance

## Abstract

Colistin-resistant (Col-R) bacteria are increasing sharply, which poses a serious threat to public health. Thymol is a phenolic compound used for its wide-spectrum antimicrobial activity, while the combination of nontraditional drugs to restore colistin activity is an attractive strategy to treat infections caused by these pathogens. This study showed that thymol could play a synergistic role with colistin against Gram-negative bacteria (GNB), including nonfermenting bacteria and Enterobacteriaceae. According to antimicrobial resistance profiles, most of the colistin-resistant strains we collected showed multidrug-resistant (MDR) phenotypes. The checkerboard method and time-kill curve confirmed the synergistic effect of thymol combined with colistin against Col-R GNB. The synergistic antibiofilm activity of thymol combined with colistin was assessed via crystal violet staining and scanning electron microscopy (SEM) assays. Results showed that compared with a single drug, the combination partially destroyed bacterial cells and inhibit the formation of bacterial biofilms. Mechanismly, the thymol/colistin combination synergistically potentiated the antibacterial activity by accelerating the damage and permeability of the bacterial outer membrane. Preliminary data indicated that the thymol/colistin combination could decrease the number of bacteria ≥2 log_10_ CFU/mL after 24 h of therapy in a mouse thigh infection model. Our results fully prove that thymol and colistin combination possesses a promising treatment option against colistin-resistant GNB infections.

**IMPORTANCE** Colistin is being considered “the last ditch” treatment in many infections caused by multidrug-resistant GNB clinical isolates, but colistin-resistant (Col-R) strains with different drug resistance mechanisms have appeared worldwide. Hence, it is of great significance to rejuvenate sensitization of clinical Col-R Gram-negative bacteria to colistin. In this study, the thymol/colistin combination showed notable antibacterial activity *in vitro* and *in vivo*. These findings suggest that the thymol/colistin combination may have promise as a treatment approach for treating the infections caused by Col-R pathogens.

## INTRODUCTION

Colistin (polymyxin E) belongs to polymyxin antibiotics, which is one of the lines of defense drugs commonly used in the treatment of multidrug resistance and carbapenem-resistant Gram-negative bacteria (GNB) infections (1), but colistin-resistant (Col-R) strains with different drug resistance mechanisms have appeared worldwide (2). In addition, colistin has dose-dependent side effects such as nephrotoxicity and neurotoxicity, which limits its clinically sufficient dose and long-term treatment options (3). In contrast, the development of novel antibiotics and their introduction into clinical use cannot keep pace with the emergence of resistant pathogens (4). To address this issue, the combination of nontraditional drugs and antibiotics has been regarded as a new treatment strategy for overcoming the drug resistance of bacteria (5).

The cell community is wrapped in the self-produced extracellular polysaccharide matrix to form a bacterial biofilm, which can enhance the adaptability of pathogens and endow them with the ability to buffer and adapt to antimicrobial agents, as an important cause of drug-resistant strains (6). Biofilms are also a major cause of chronic and equipment infections (7), placing a heavy burden on the health care system. Biofilm-related bacterial infections are recognized as being exceedingly difficult to treat, thus validating the promising strategy of seeking to inhibit bacterial adhesion before biofilm formation.

Thymol is extracted from the spice oregano, as a medicinal plant essential oil. It has attracted wide attention because of its anti-inflammatory, antioxidant, antibacterial, and antifungal biological activities (8–10). In recent years, thymol has been attracting the close attention of researchers, as monoterpene alcohol, its antibacterial activity is related to its structure, thymol can overcome the lipid barrier and target the cell membrane of the hydrophobic pathogen, which can be integrated into the lipid layer of the cell membrane and induce its instability. It was accepted by the European Commission and the United States Food and Drug Administration (FDA) for safety to the consumer and plays roles as synthetic flavoring (21 CFR 172.515), preservative and indirect food additive of adhesives (21 CFR 175.105) for a long time (11).

It is interesting to explore the combined effect of thymol since thymol has strong membrane-perturbing capacities. However, there is a lack of studies on the synergistic activity of thymol combined with colistin in the treatment of Col-R GNB. Therefore, the main purpose of this study is to determine the synergistic activity and antibiofilm formation effect of colistin and thymol combination, providing new possible therapeutic strategies to overcome the infections of colistin resistance in the future.

## RESULTS

### Antimicrobial susceptibility assay.

A total of 32 nonduplicated Gram-negative Col-R strains were randomly selected. Most of the strains show MDR phenotypes. On the whole, colistin MICs of the Col-R strains ranged from 4 to 1024 mg/L, while thymol MICs of *Enterobacteriaceae* strains were 128 to 256 mg/L and 512 to 2048 mg/L for P. aeruginosa (Table 1).

**TABLE 1:** The MICs of commonly used clinical antibiotics and thymol against Col-R GNB

Species	Strains^*a*^	Antibiotics^*b*^									Thymol
		ATM	CAZ	FEP	IPM	CIP	LVX	GEN	TOB	COL	
		Breakpoints(S-R)^*c*^ MIC (mg/L)									
		8-32	8-32	8-32	2-8	0.5-2	1-4	4-16	4-16	2-4	
P. aeruginosa	TL1671	8	4	8	2	0.25	1	2	1	32	1,024
	**TL1736**	4	4	2	16	1	1	32	8	8	512
	**TL1744**	32	32	16	16	32	8	≥256	32	4	1,024
	TL2314	16	32	16	4	0.5	2	8	2	8	1,024
	**TL2917**	32	16	16	16	0.25	2	8	8	8	2,048
	**TL2967**	128	16	32	16	8	16	8	8	4	1,024
	**TL3008**	4	2	4	16	0.5	1	16	4	4	1,024
	**TL3086**	128	16	16	≥256	16	8	≥256	128	16	1,024
		4–16	4–16	2–16	1–4	0.25–1	0.5–2	4–16	4–16	32	
E. coli	**DC90**	≥256	32	64	≥256	64	32	≥256	128	4	256
	**DC3737**	≥256	≥256	≥256	128	≥256	≥256	≥256	≥256	16	256
	**DC3806**	64	64	16	1	4	8	16	16	4	256
	**DC3846**	128	64	≥256	0.5	≥256	128	≥256	64	8	256
	**DC4887**	1	4	32	1	4	16	16	8	8	128
	**DC5262**	≥256	≥256	≥256	4	2	16	≥256	≥256	8	256
	**DC5286**	≥256	128	≥256	0.25	128	64	4	4	8	256
	**DC7333**	≥256	≥256	≥256	16	≥256	128	128	≥256	4	256
K. pneumoniae	**FK20**	≥256	128	≥256	16	≥256	64	8	≥256	4	256
	**FK150**	0.0125	0.5	4	0.5	8	128	128	≥256	8	256
	**FK169**	1	16	0.5	4	2	1	1	64	≥64	256
	**FK1342**	128	≥256	≥256	0.25	1	0.5	1	4	≥64	256
	FK1986	0.0125	0.25	0.0125	0.25	0.0125	0.025	2	1	16	256
	**FK3810**	0.0125	128	≥256	32	≥256	128	≥256	≥256	≥64	128
	**FK6663**	≥256	≥256	≥256	32	≥256	≥256	≥256	≥256	32	256
	**FK6696**	≥256	64	≥256	128	≥256	64	≥256	≥256	≥64	128
*E. cloacae*	CG648	16	4	4	4	0.25	0.25	≤1	≤0.25	≥64	256
	**CG737**	≤1	2	≤1	0.5	≥4	≥8	≤1	8	≥64	256
	**CG741**	≤1	2	≤1	0.25	≥4	≥8	≤1	8	≥64	256
	**CG884**	≥64	≥64	≤1	0.25	0.25	1	≤1	≤1	≥64	256
	**CG934**	≥64	≥64	≤1	0.25	≤0.25	0.25	≤1	≤1	≥64	128
	**CG1050**	≥64	≥64	≤1	0.5	1	1	≤1	≤1	≥64	256
	**CG1051**	≥64	≥64	2	0.25	1	1	≤1	≤1	≥64	256
	**CG1479**	≥64	≥64	8	16	≤0.25	0.25	≤1	≤1	≥64	256

*^a^*Boldface strain number indicates multidrug resistant (MDR) strain.

*^b^*GNB, Gram-negative bacteria; ATM, aztreonam; CAZ, ceftazidime; FEP, cefepime; IMP, imipenem; CIP, ciprofloxacin; LVX, levofloxacin; GEN, gentamicin; TOB, tobramycin; COL, colistin.

*^c^*S-R represents the susceptible (S) breakpoint to resistant (R) breakpoint, according to CLSI supplement M100 (30th edition) and EUCAST.

### Evaluation of synergy by checkerboard assays.

According to the checkerboard assay, there was a significant decrease in MIC values in different combinations of thymol with colistin. The antibacterial effects of colistin combined with thymol on Col-R E. coli (*n* = 8), K. pneumoniae (*n* = 8), E. cloacae (*n* = 8), and P. aeruginosa (*n* = 8) were demonstrated by checkerboard assay. Checkerboard assays showed that 30 strains (30/32) showed a significant synergistic effect (defined as a fractional inhibitory concentration index [FICI] of ≤ 0.5), which meant the broad-spectrum antibacterial effect of the combination on GNB infection. Additionally, the colistin MICs of all Col-R strains in the presence of thymol were reduced to ≤ 1 mg/L (Table 2).

**TABLE 2:** FICI value for colistin/thymol combinations against Col-R Gram-negative

Species	Strains	Monotherapy (mg/L)		Combination (mg/L)		FICI	Interpretation
		Colistin	Thymol	Colistin	Thymol		
E. coli	DC90	4	256	0.125	64	0.28	Synergistic
	DC3737	16	256	0.5	32	0.155	Synergistic
	DC3806	4	256	0.125	64	0.28125	Synergistic
	DC3846	8	256	0.5	32	0.1875	Synergistic
	DC4887	8	128	0.125	64	0.515	Synergistic
	DC5262	8	256	0.06	64	0.2575	Synergistic
	DC5286	8	256	0.06	64	0.2575	Synergistic
	DC7333	4	256	0.125	64	0.28	Synergistic
K. pneumoniae	FK20	4	256	0.125	128	0.53	No interaction
	FK150	8	256	0.25	32	0.156	Synergistic
	FK169	≥64	256	1	32	0.14	Synergistic
	FK1913	≥64	256	0.25	64	0.254	Synergistic
	FK1986	16	256	0.125	32	0.133	Synergistic
	FK3810	≥64	128	0.25	16	0.129	Synergistic
	FK6663	32	256	0.125	64	0.254	Synergistic
	FK6696	≥64	128	0.25	64	0.504	No interaction
*E. cloacae*	CG648	≥256	256	0.125	32	0.125	Synergistic
	CG737	≥256	256	0.125	32	0.125	Synergistic
	CG741	128	256	0.25	32	0.127	Synergistic
	CG884	≥512	256	0.25	32	0.061	Synergistic
	CG1050	256	256	0.25	32	0.127	Synergistic
	CG1051	≥256	256	0.25	32	0.126	Synergistic
	CG1479	≥512	256	0.5	64	0.256	Synergistic
	CG1574	≥256	256	0.25	32	0.126	Synergistic
P. aeruginosa	TL1671	32	1,024	1	64	0.0937	Synergistic
	TL1736	8	512	0.25	128	0.28	Synergistic
	TL1744	4	1,024	0.25	64	0.125	Synergistic
	TL2314	8	1,024	1	64	0.1875	Synergistic
	TL2917	4	2,048	0.25	128	0.125	Synergistic
	TL2967	4	1,024	0.25	128	0.188	Synergistic
	TL3008	16	1,024	0.125	256	0.258	Synergistic
	TL3086	32	1,024	0.5	128	0.141	Synergistic

### Time-kill assays.

**FIG 1:**
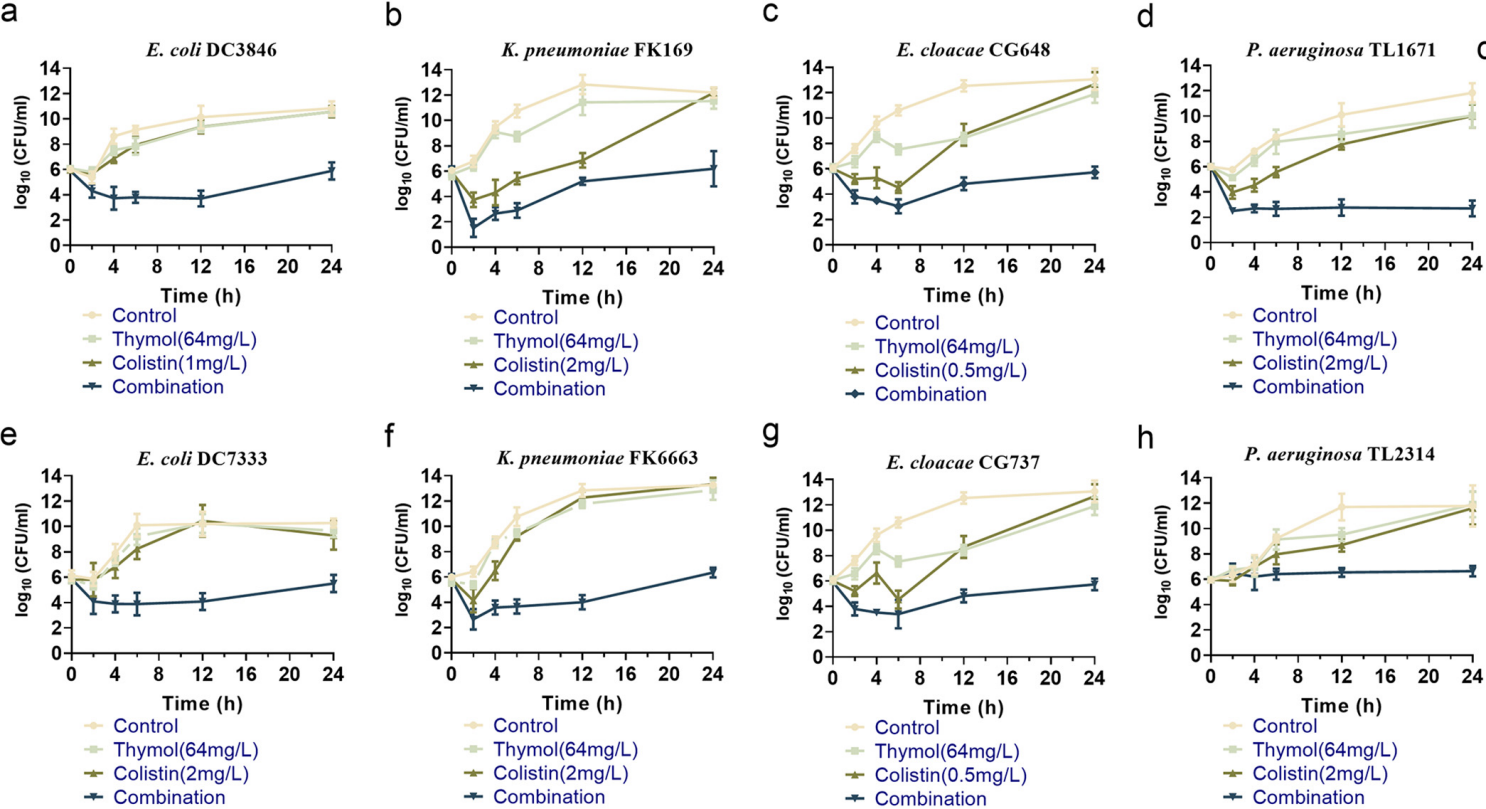
Time-killing curves of colistin and thymol alone or in combination against Col-R GNB. (a, e) Colistin resistance E. coli; (b, f) colistin resistance *K. pneumonia*; (c, g) colistin resistance E. cloacae; (d, h) colistin resistance *P. aeruginosa*.

To study the effect of this combination on the growth kinetics of Col-R Gram-negative bacteria, a time-kill assay was conducted in randomly selected Col-R E. coli DC3846, E. coli DC7333, K. pneumoniae FK169, K. pneumoniae FK6663, E. cloacae CG648, E. cloacae CG737, and P. aeruginosa TL1671, P. aeruginosa TL2314. The drug concentrations used for the time-kill curve were derived from checkerboard results, with FICI < 0.5. The concentrations of thymol that were selected included 32 and 64 mg/L, and the concentrations of colistin were 0.5,1 and 2 mg/L. As shown in Fig. 1, for the initial growth phase, the thymol monotherapy treatment also showed good inhibition on several of the strains (K. pneumoniae FK169 and both E. cloacae strains), and the growth of E. cloacae strains were still inhibited by thymol at 64 mg/L even at 12 h. While the CFU increased from 6 to 24 h for all strains with colistin monotherapy treatment, and none of monotherapy groups inhibited the growth of bacteria when the cultures reached the midlog growth phase. The combination of colistin and thymol showed good synergistic and bactericidal activity against all tested strains within 24 h. The combination of colistin and thymol resulted in the regrowth of 2 P. aeruginosa being inhibited from 4 h. In a word, the combination of colistin and thymol enhanced their killing activity against drug exposure.

### Impact of the combination of colistin with thymol on biofilm formation.

**FIG 2:**
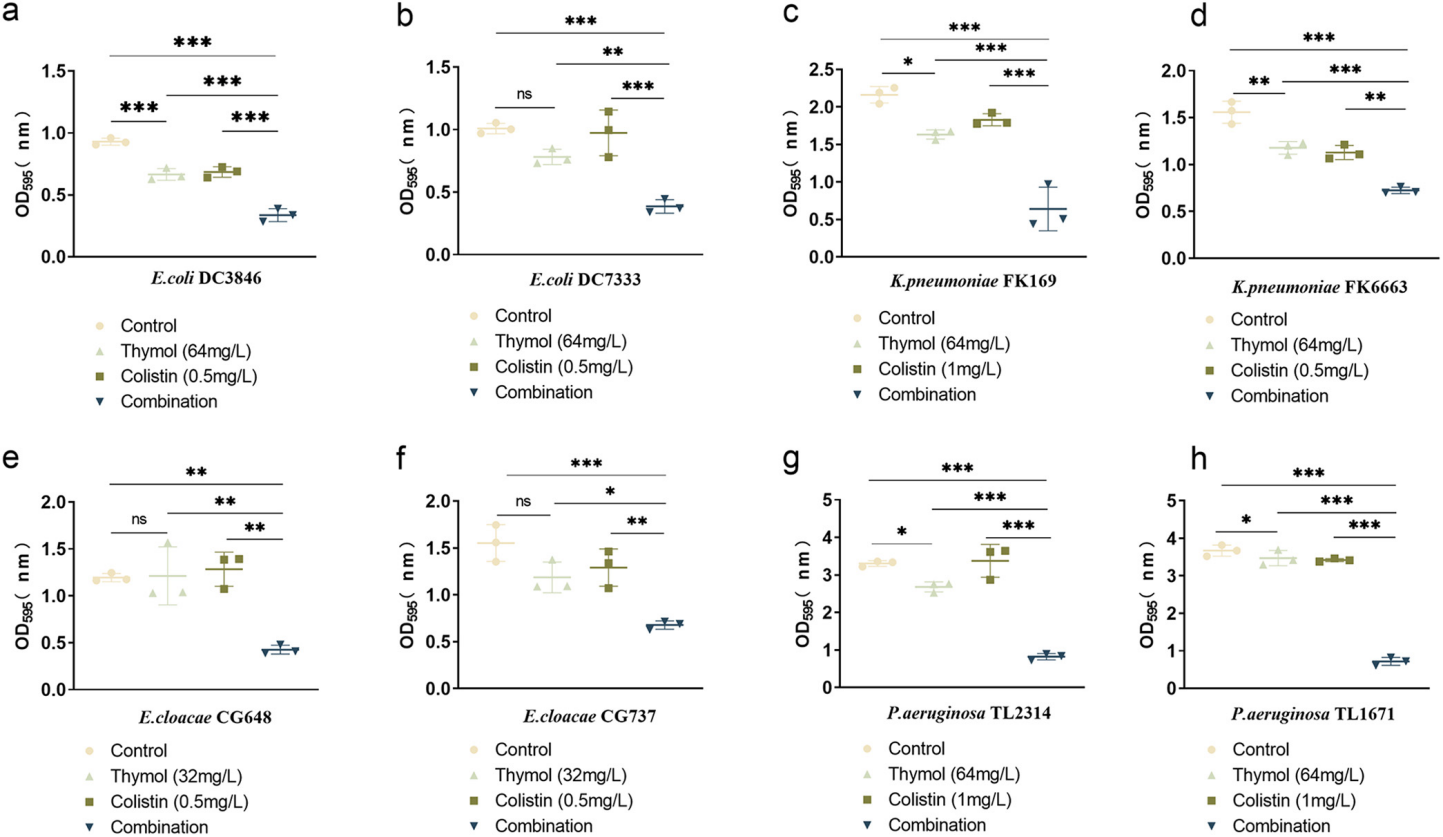
Biofilm inhibitory effects of colistin combined with thymol on Col-R GNB. ns, not statistically significant, *P < *0.05 (*), *P < *0.01 (**), and *P < *0.001 (***). Data were analyzed using One-Way ANOVA followed by a Tukey's *post hoc* test. The experiments were performed three times. Data are expressed as mean ± standard deviation. GNB, Gram-negative bacteria.

The effect of thymol combined with colistin on biofilm formation was studied after crystal violet staining. As shown in Fig. 2, the thymol monotherapy treatment also showed good inhibition on several of the strains (5/8). And compared with the single-agent group, thymol-colistin combination effectively inhibited bacterial biofilm formation, which may be due to the decreased viability of cultures (*P > *0.05).

### Scanning electron microscopy (SEM).

**FIG 3:**
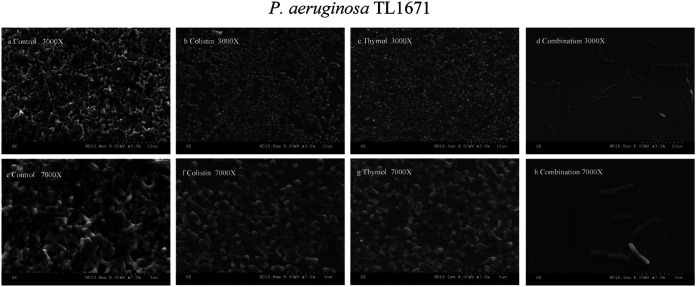
SEM images of Col-R P. aeruginosa TL1671 after treatment with 2 mg/L colistin alone (b, f), 64 mg/L thymol alone (c, g), or combination (d, h) for 2h. (a, e) represent the control condition.

**FIG 4:**
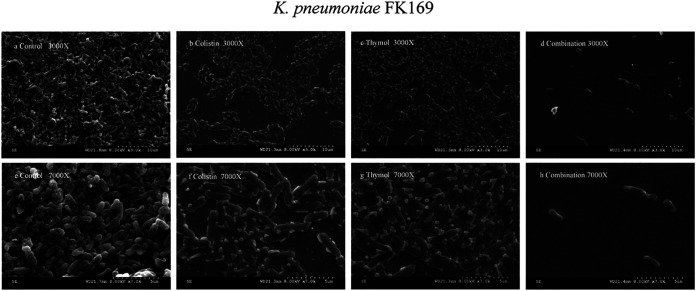
SEM images of Col-R *K. pneumonia* FK169 after treatment with 1 mg/L colistin alone (b, f), 32 mg/L thymol alone (c, g), or combination (d, h) for 2h. (a, e) represent the control condition.

The structure of biofilm affected by thymol and colistin was assessed by SEM (Fig. 3 and 4). SEM images showed a large number of untreated bacterial cell biofilms covering the whole field of vision, with bacteria interwoven in the interior, with a magnification of ×3000 (Fig. 3a and 4a). Biofilms treated with colistin (2 mg/L) and thymol (64 mg/L) alone also formed biofilms (Fig. 3b and c, 4b and c), and a large number of cells were morphologically intact. However, the samples treated with thymol combined with colistin resulted in a significant reduction of cells, biofilm number and density, and a small amount of bacterial aggregation. In general, at 7000× magnification, the control group and the single-drug group showed complete morphology and dense arrangement (Fig. 3e and f, 3g, 4e, 4f and g), while the structure of biofilm treated with the combination destroyed and the number was decreased (Fig. 3h and 4h).

### Mechanisms for drug synergy.

**FIG 5:**
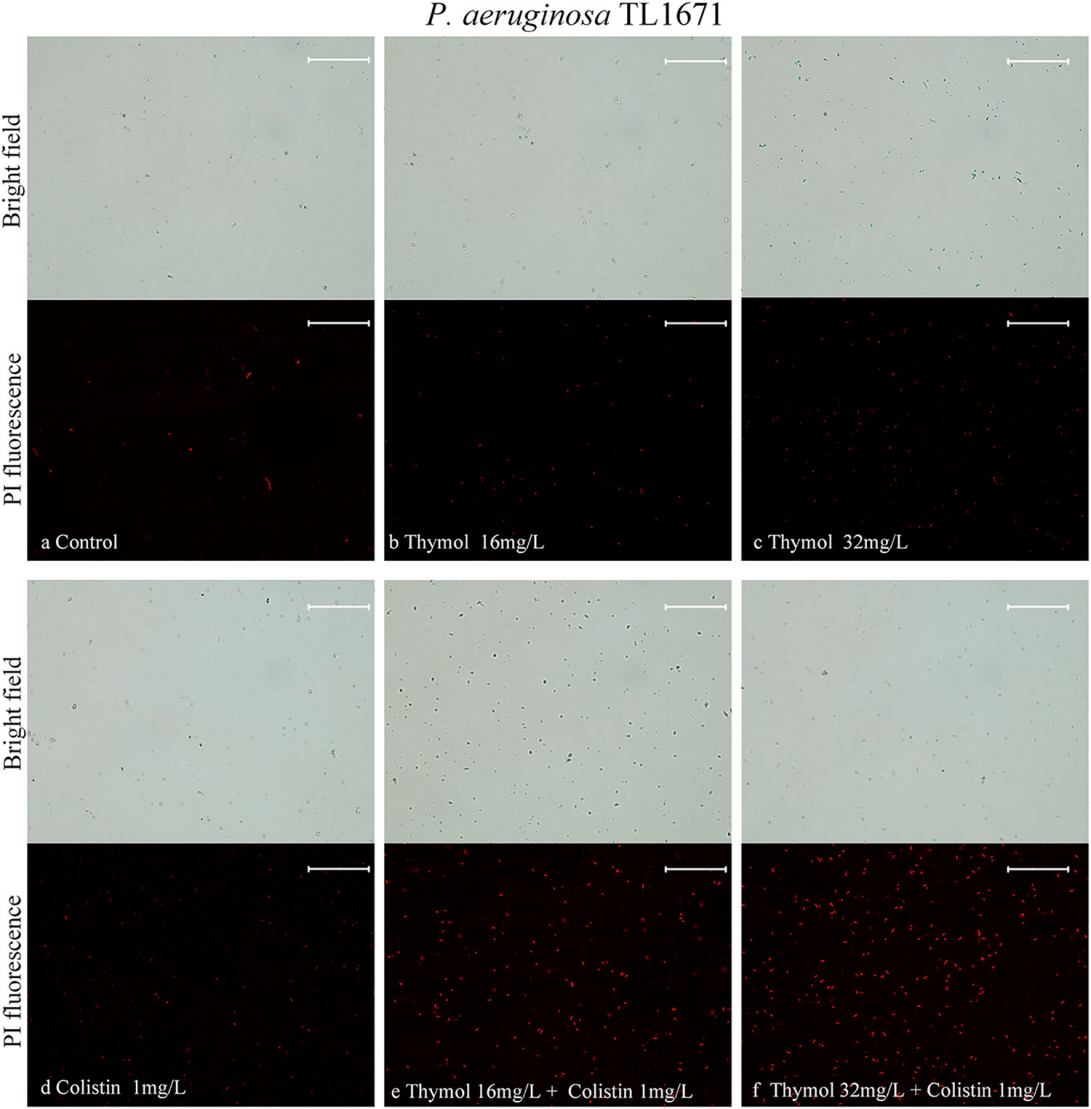
Fluorescence microscopy imaging of exponential-phase P. aeruginosa TL-1671, which were treated with thymol and colistin alone or in combination and incubated with 50 mg/L PI for 10 min before imaging. (a) LB broth control; (b-c) cells treated with thymol at 16 mg/L, 32 mg/L; (d) cells treated with colistin with 1 mg/L; (e-f) cells exposed to a combination of thymol and colistin.

**FIG 6:**
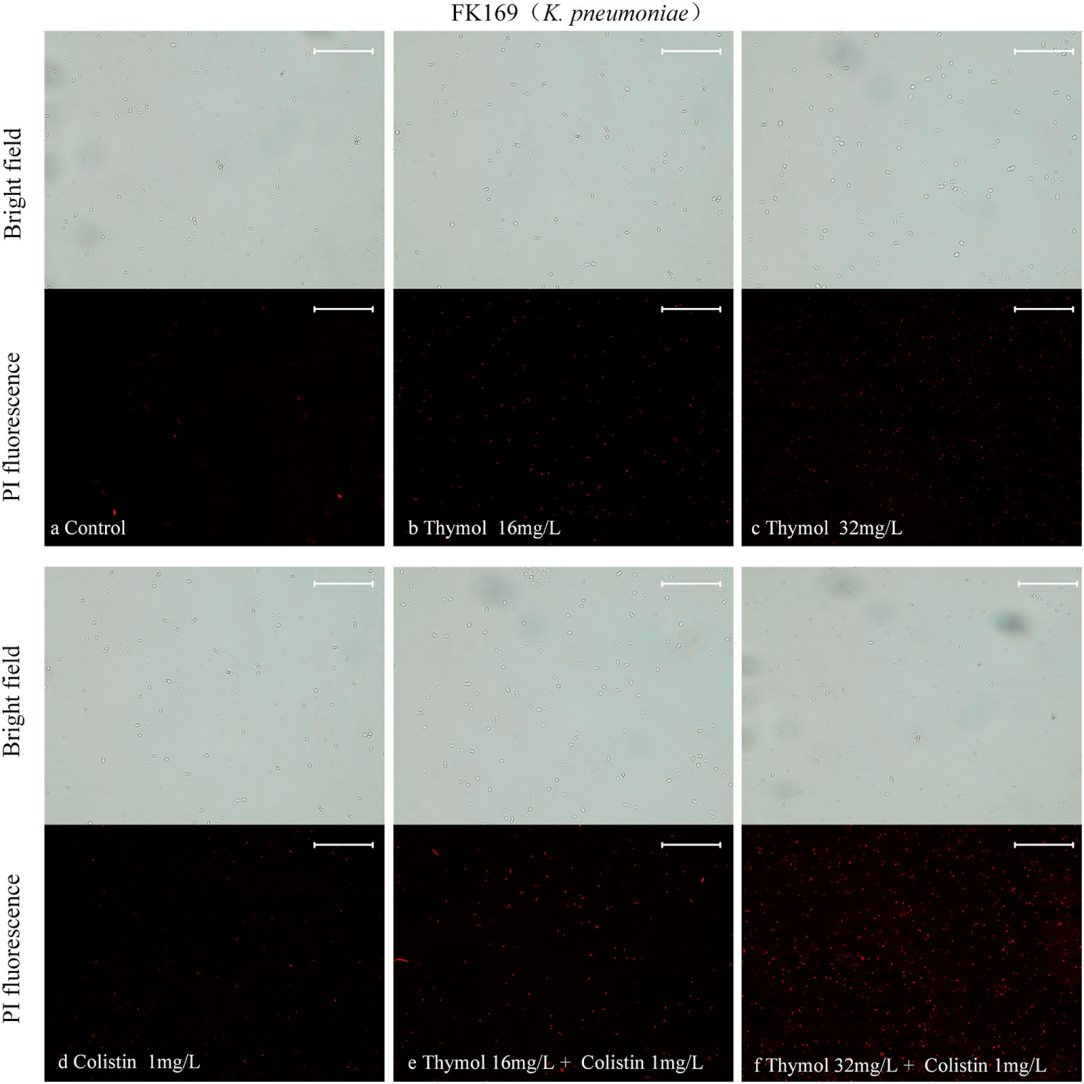
Fluorescence microscopy imaging of exponential-phase *K. pneumonia* FK-169, which were treated with thymol and colistin alone or in combination and incubated with 50 mg/L PI for 10 min before imaging. (a) LB broth control; (b-c) cells treated with thymol at 16 mg/L, 32 mg/L; (d) cells treated with colistin with 1 mg/L; (e-f) cells exposed to a combination of thymol and colistin.

**FIG 7:**
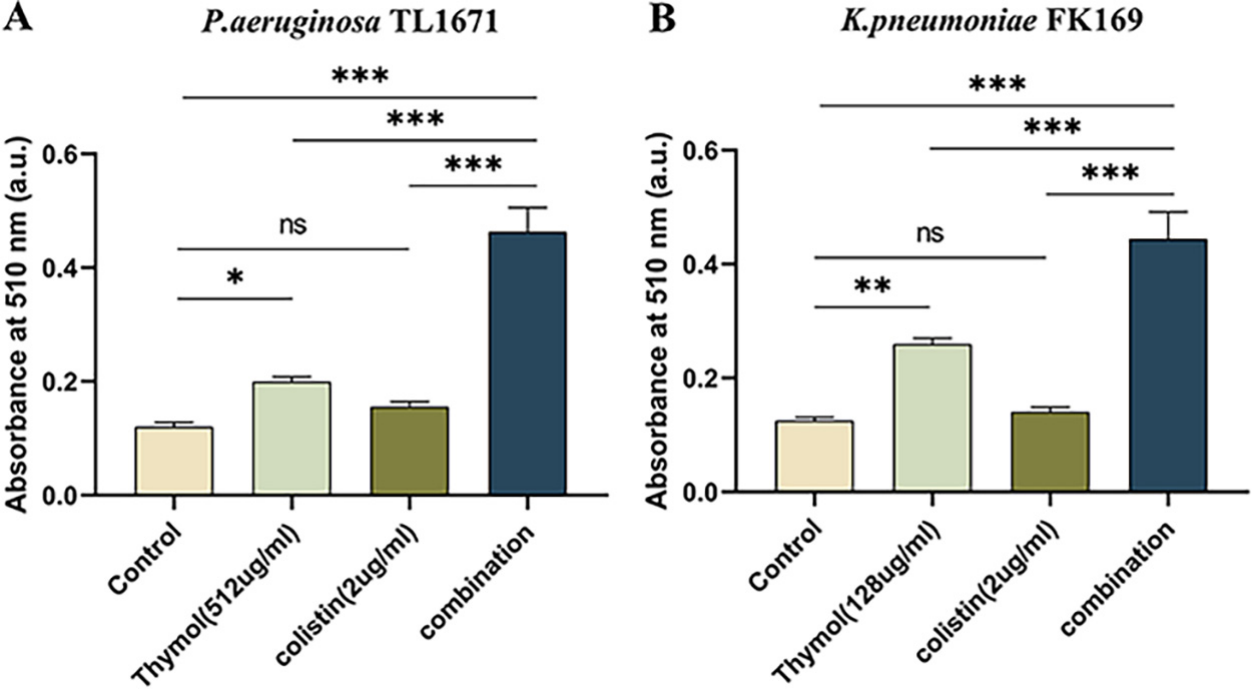
Combination of thymol and colistin contributes to the leakage of bacterial alkaline phosphatase (ALP), the absorbance was measured at 510 nm. The results are shown as the mean and standard deviation of three independent experiments. ns stands for not significant, *, *P < *0.05; **, *P < *0.01; ***, *P < *0.001, data were analyzed using One-Way ANOVA followed by a Tukey's *post hoc* test.

We evaluated the cell membrane permeability of P. aeruginosa TL1671 and K. pneumoniae FK169 using propidium iodide (PI) staining. As revealed by fluorescence microscopic analysis (Fig. 5, 6), When pre-incubation of the cell with colistin at 0.5 mg/L or 1 mg/L, little effect on cell membrane permeability was observed. However, pre-incubation of the cells with thymol resulted in a concentration-dependent enhance in fluorescence intensity due to PI uptake and DNA binding, which indicated that the integrity of the cell membrane gradually decreased. In addition, the cell membrane permeability induced by thymol and colistin alone or in combination was also evaluated by measuring the leakage of alkaline phosphatase (ALP). As shown in Fig. 7, the colistin/thymol combination promotes stronger extracellular ALP signals compared to colistin treatment alone in all the tested isolates. Taken together, thymol may enhance the bactericidal effect of colistin by enhancing outer membrane permeability.

### In vivo treatment verification.

**FIG 8:**
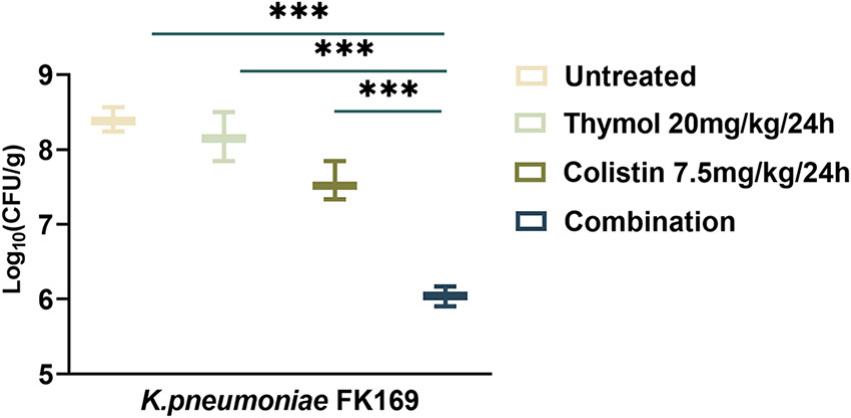
Quantified log_10_ CFU/thigh in mice at 24 h posttreatment of various treatments. Changes in mouse thigh muscles (Δlog_10_ CFU/thigh) after 24 h of monotherapy or combination therapy using different dosing regimens against colistin-resistant strain of K. pneumoniae FK 169 (*n* = 6).

The efficacy of thymol in combination with colistin was further validated *in vivo* by the mouse thigh infection model. The results of the same are shown in Fig. 8 K. pneumoniae FK169 was randomly selected as experimental strain. Thymol at 20 mg/kg and colistin at 7.5 mg/kg lightly inhibited K. pneumoniae FK169 after injection of 24 h. However, thymol combination with colistin showed higher efficacies than those of single treated (*P < *0.05). Indicating that the combination of the two drugs to colistin-resistant K. pneumoniae FK169 has a significant synergistic antibacterial effect.

## DISCUSSION

In recent years, the widespread use of antibiotics to treat bacterial infections has led to the emergence of MDR bacteria, which pose a great hazard to public health (12). With the lack of new antibiotics in the drug discovery pipeline to treat Gram-negative infections, coupled with accelerated evolution of antibiotic resistance, colistin is recognized as the last-resort antibiotic for the treatment of infections caused by MDR GNB, however, its use for the treatment of infections due to MDR bacteria, such as P. aeruginosa, and *Enterobacteriaceae*, has contributed to the emergence of colistin resistance (13). Hence, the development of effective and broad-spectrum antibacterial strategies is urgently required to prevent and treat Col-R GNB infections. In this study, we evaluated the potential application of colistin combined with thymol in the treatment of Col-R isolates infection.

Thymol is believed to act by increasing the permeability of cell membranes (14). This process is accompanied by an increase in permeability and a decrease in the activity of intimal proteins (such as enzymes and receptors) (9). Although thymol has cytotoxicity at high concentrations, it has no toxicity at common doses and can be considered a safe drug (15). The *in vitro* and *in vivo* cytotoxicity of thymol was assessed by Robledo et al., 2005. In comparison with the low concentrations used in our study, for thymol LD50 value of 400 mg/L using U-937 human promonocytic cells has been reported. With an oral dose of 40 mg/kg of body weight/day, thymol was not toxic to golden hamsters based on corporal weight, behavior and serum levels of bilirubin, uric acid, and glucose (16). In addition, the toxicity evaluation of thymol in mice was also reported to be safe and nontoxic, no changes in mic The *in vitro* and *in vivo* e behavior, mobility, and feeding habits were observed (17).

Previous studies indicated that thymol may be promising adjuvants to other antimicrobials against pathogens due to its potent penetrability. Some previous articles reported that thymol combined with other antibiotics such as vancomycin and ciprofloxacin had certain antibacterial activity against S. aureus and K. pneumoniae (18, 19), but there was no study on thymol and colistin combination against Col-R GNB.

In this work, we first reported the synergistic activity of thymol combined with colistin to Col-R GNB, including nonfermentative bacteria (P. aeruginosa) and *Enterobacteriaceae* (E. coli, K. pneumoniae, E. cloacae), to comprehensively evaluate and reveal a potential strategy against Col-R strains. The results of antimicrobial susceptibility showed that most of the strains showed multidrug resistance phenotype (Table 1), indicating that we should timely monitor the situation of colistin resistance to effectively prevent and control the emergence and spread of resistant strains. Checkerboard assays were used to determine the synergistic effect between drugs. We found that the FICI of thymol and colistin in the Col-R GNB was less than 0.5 (except K. pneumoniae FK20 and K. pneumoniae FK6696, additive effect) (Table 2). Previous work has reported a potential antibacterial effect of thymol (10), however, a comparison of antibacterial activity among P. aeruginosa and *Enterobacteriaceae* is rarely reported. This study combined with the work of Wattanasatcha et al. (10) suggests that the *Enterobacteriaceae* strains showed higher sensitivities to thymol than P. aeruginosa, which is in line with the SEM results.

Time killing curve showed that thymol/colistin combination had a significant synergistic effect in the tested strains. In addition, the combination of thymol and colistin can effectively inhibit the formation of bacterial biofilm. The SEM results showed that compared with the single drug group, the number of biofilm cells in the thymol combined with colistin group decreased significantly. Thymol has been reported to inhibit biofilm formation at sub-MICs (20, 21). And exploring the antibiofilm mechanism of combination is worth further investigation.

Rapid penetration of antibiotics is a main factor affecting bactericidal activity, and effective permeabilization of the outer membrane may overcome intrinsic resistance pathways. In previous studies (22), thymol-induced cell membrane damage against fungus was indicated by PI staining, based on this, we further explored the mechanism of synergy action via PI staining and alkaline phosphatase assay. The results indicated that thymol can increase membrane permeability to overcome colistin resistance.

Most of the patients who were infected with Col-R GNB were critically ill patients with ICU, which often showed low immunity (23). In combination with thymol could not only reduce the risk of nephrotoxicity but also its anti-inflammatory and antiviral properties and therapeutic potential for metabolic diseases are also of great benefit to clinical treatment (24–26). In previous studies, the *in vivo* antibacterial activity of thymol was confirmed in mouse model experiments (27, 28). Thus, we further constructed the mouse thigh infection model and evaluated the antimicrobial efficacy of thymol/colistin combination *in vivo*. Notably, colistin combined with thymol can significantly reduce the number of bacteria in mice.

The synergistic effects of thymol and antibiotics lead to new clinical choices via outer membrane destabilization, which reduces the toxicity of colistin, avoiding the emergence of resistant variants that might otherwise arise during treatment. The currently developed Solid formulation techniques on the improved bioavailability and activity of nanocapsules of thymol (e.g., microencapsulation, nanoparticles, and liposomes) are also helpful to their controlled release and targeted delivery of thymol (21, 29–35).

### Conclusion.

Taken together, this is the first report of synergistic activity of colistin in combination with thymol in Gram-negative bacteria, and our data revealed that the combination of thymol, as a potent permeabilizer, and colistin represents a new therapeutic strategy for a bacterial infection to meet current clinical challenges via teaching an old dog a new trick.

## MATERIALS AND METHODS

### Bacterial strains and chemicals.

A total of 32 nonduplicated Col-R GNB (table S1) were isolated from the First Affiliated Hospital of Wenzhou Medical University in China, including Col-R Escherichia coli (E. coli) (*n* = 8), Klebsiella pneumoniae (K. pneumoniae) (*n* = 8), Enterobacter cloacae (E. cloacae) (*n* = 8) and Pseudomonas aeruginosa (P. aeruginosa) (*n* = 8). These strains were all identified by matrix-assisted laser desorption/ionization time-of-flight mass spectrometry (MALDI-TOF/MS; bioMérieux, Lyons, France), E. coli ATCC25922, and P. aeruginosa ATCC27853 were purchased from the national center of the clinical laboratory (NCCL) and used as control. All antibiotics used in this study, including colistin, aztreonam, ceftazidime, cefepime, imipenem, ciprofloxacin, levofloxacin, gentamicin, tobramycin, and amikacin were purchased from Wenzhou Kangtai Biological Technology Co., Ltd. (Zhejiang, China). Solvents and diluents for the preparation of antibiotics complied with the latest guidelines published by the Clinical and Laboratory Standards Institute (CLSI 2020). Thymol was purchased from commercial suppliers (Sigma-Aldrich, Saint Louis, USA), and was dissolved in dimethyl sulfoxide (DMSO) (Sigma-Aldrich, Saint Louis, USA), the concentration of ≤1% (vol/vol) (36).

### Determination of antimicrobial susceptibility.

MICs of colistin and thymol were determined by cationic adjusted Mueller-Hinton broth (CAMHB) microdilution method (37), respectively. Colistin or thymol was twice diluted; serial 2-fold dilutions ranging from 128 to 0.0625 mg/L for colistin, and 1024 to 0.5 mg/L for thymol were added in CAMHB 96-well microtiter plates. A final bacterial suspension of 7.5 × 10^5^ CFU/mL was added to each well and incubated with colistin or thymol at 37°C for 16 to 20 h. Growth of cells in the plate was determined by visual inspection after 16 to 20 h of incubation at 37°C. The MIC was defined as the lowest concentration that inhibited visible growth of the tested isolate. The interpretation of antimicrobial susceptibility assay is based on the breakpoint point of antibiotics provided by CLSI 2020 (38). The breakpoints proposed by CLSI were used for colistin (susceptible, ≤2 mg/L; resistant, ≥4 mg/L), each MICs test was verified duplicate.

### Checkerboard assays.

The synergistic activity between colistin with thymol was determined through a checkerboard assay described in the previous study (39). Briefly, the drugs were arrayed in serial concentrations and mixed to create different concentration combinations. The rest of the procedure involving the addition of bacteria and measurement of growth was carried out as described in the previous section for measurement of MICs. For the checkerboard assay using a microdilution approach in a 96-well plate, minimum concentrations of the compounds that inhibited visible growth of bacteria after 16 to 20 h were chosen as the MICs. The Fractional Inhibitory Concentration Index (FICI) for two compounds A and B is defined by the following equation: FICI = FICA + FICB = (CA/MICA) + (CB/MICB), where MICA and MICB are the MICs of drugs A and B alone, respectively, and CA and CB are the concentrations of the drugs in combination, respectively, in all of the wells corresponding to a MIC. (Interactions were interpreted as follows: synergy for FICI ≤ 0.5; no interaction for 0.5 < FICI ≤ 4; antagonism for FICI > 4) (40).

### Time-kill assays.

To study the effect of this combination on the growth kinetics of Col-R Gram-negative bacteria, a time-kill assay was conducted in randomly selected Col-R E. coli (*n* = 2), K. pneumoniae (*n* = 2), E. cloacae (*n* = 2), and P. aeruginosa (*n* = 2) strains, as previously described (41). Briefly, bacteria were incubated with thymol and colistin alone or in combinations at 1 × 10^6^ CFU/mL. Tubes containing LB alone served as the negative control. The thymol monotherapy group with a final thymol concentration of 32 to 64 mg/L, the colistin monotherapy group with a final colistin concentration of 0.5 to 2 mg/L, and the monotherapy concentration of the corresponding strain was added to the combined group. The bacterial suspensions were incubated at 37°C with moderate shaking. CFU/were enumerated on MHA agar plates at 0, 2, 4, 6, 12, and 24 h.

Synergistic activity was defined as a ≥ 2 log_10_ decrease in CFU/mL of the combination compared to the most active monotherapy.

The Col-R E. coli (*n* = 2), K. pneumoniae (*n* = 2), E. cloacae (*n* = 2), and P. aeruginosa (*n* = 2) strains we used for biofilm formation inhibition assays were based on the results of the checkerboard assay. The biofilm formation inhibition assays were performed as mentioned above with some modifications (42). Bacteria were inoculated on plates and cultured overnight. The bacterial suspension was adjusted to 0.5 McFarland and diluted 1:100 in fresh LB broth before being added to a 96-well plate. Thymol and colistin were then added to a 96-well plate at a final concentration of 32 to 64 mg/L and 0.5 to 1 mg/L, respectively, in combination and alone. The 96-well plates were then incubated at 37°C for 24 h. After 24 h of incubation, We washed the 96-well plate twice with 200 μL of 1× PBS (Sigma-Aldrich, Milan, Italy) to remove the planktonic bacteria. The stable biofilm quality was assessed using crystal violet staining (43). Read the absorbance at 595 nm on a microplate reader (MultiskanFC) (44). The experiment was repeated three times. *P* value < 0.05 was considered significant.

### Scanning electron microscope.

SEM was employed to examine the effect of the colistin–thymol combination on the biofilms of P. aeruginosa TL1671 and K. pneumoniae FK169. 1800 μL of the LB broth and 200 μL of bacterial solution were added to the wells of a 6-well plate containing sterilized glass coverslip (lot number: NO.10211818C, CITOGLAS Co., Ltd., China) and they were first grown to midlog phase in LB. Afterward, coverslips were washed with aseptic PBS 2 times. Biofilm coverslips were divided into four groups, including the control group, thymol monotherapy group, colistin monotherapy group, and combination group.LB broths (2000 μL) in the final concentrations of 2 mg/L colistin or 64 mg/L thymol was added to the monotherapy group, and LB broth (2000 μL) containing a final concentration of 2 mg/L colistin and 64 mg/L thymol was added to the combined group and incubated at 37°C under static conditions for 24 h. After incubation, planktonic bacteria were washed away with sterile PBS 2 times and fixed biofilms with 2.5% glutaraldehyde (Merck) at 4°C for 2 h, followed by the dehydration in several stages with a serial dilution of ethanol (30%, 50%, 70%, 90% and 100% vol/vol) for 15 min each and air-dried at room temperature in a desiccator connected to a vacuum. After that, the sample was placed into the ion sputtering instrument and gold was sprayed with a thickness of 5 nm. SEM observations were performed at 10 kV with a Hitachi S3000N (Tokyo, Japan) according to the manufacturer's instructions (45).

### Permeability of cell membranes.

Cell membrane permeability was examined as described previously with modifications (46). Exponential-phase cells of P. aeruginosa TL1671 and K. pneumoniae FK169 were treated with the test drug alone (thymol 16, and 32 mg/L; colistin 1 mg/L) or in combination for 2 h and incubated at room temperature in PI (50 mg/L) for 10 min. Bright-field and fluorescent images were obtained using a fluorescence microscope (Nikon Eclipse 80i, Nikon, Tokyo, Japan) with red filters to visualize PI stained cells, equipped with a Nikon DS-Ri2 high-definition color digital camera and the Nikon software NIS-elements F imaging software. For the ALP assay, P. aeruginosa TL1671 and K. pneumoniae FK169 were inoculated into a liquid medium with four different conditions as blank control, thymol (512 mg/L or 128 mg/L) colistin (2 mg/L), and thymol-colistin combination. The samples were incubated at 37°C with shaking at 180 rpm for 24 h. After 24 h, the suspension was then centrifuged at 5000 rpm for 3 min. The supernatant was collected and further clarified by second centrifugation, and the activity of ALP was determined with an ALP assay commercial kit (47). All tests were performed in triplicate.

### In vivo evaluation of synergy in the mice infection model.

The neutropenic mouse thigh infection model was constructed for *in vivo* studies. Female BALB/c mice, 5 to 6 weeks old (Charles River, Hangzhou, China), were used in this experiment. Mice were maintained following the National Standards for Laboratory Animals of China (GB 14925–2010). All animal studies were approved by the Zhejiang Association for Science and Technology SYXK [ID: SYXK (Zhejiang) 2018-0017] and conducted in accordance with Wenzhou Laboratory Animal Welfare and Ethics guidelines.

In short, Female BALB/c mice were rendered neutropenic by cyclophosphamide (two consecutive doses of 150 and 100 mg per kg delivered on 4 and 1 days before infection). A colistin-resistant strain (K. pneumoniae FK169) was randomly selected, and 100 μL of exponentially growing bacterial suspension of 1.5 × 10^7^ CFU/mL were injected into each posterior thigh muscle. 2 h after bacterial inoculation, colistin was administered intraperitoneally at 7.5 mg/kg every 24 h, as monotherapy or in combination with thymol (20 mg/kg every 24 h) (48). The mice were euthanized after 24 h of therapy and untreated control mice. Bacterial burden was quantified by CFU counts from posterior thigh homogenates. Groups of three mice (6 thigh infections) were included in each dosing regimen. The thighs were aseptically removed, weighed, homogenized, serially diluted, and plated on Trypticase soy agar for CFU titers.

### Statistical analysis.

The results regarding the biofilm formation were expressed as mean values ± standard deviation. The statistical significance of differences between controls and experimental groups was evaluated by using one-way ANOVA with *post hoc* analysis using Tukey's test to evaluate significant differences between groups. For all analyses: *, *P < *0.05; **, *P < *0.01; ***, *P < *0.001; ns *P > *0.05.
