# Supplementary material for: Thymol Increases Sensitivity of Clinical Col-R Gram-Negative Bacteria to Colistin
Source: Microbiol Spectr. 2022 Jun 14;10(4):e00184-22. doi: 10.1128/spectrum.00184-22 (PMC9431615; doi:10.1128/spectrum.00184-22)
Supplement: Supplemental file 1 — Supplemental material. Download spectrum.00184-22-s0001.pdf, PDF file, 0.1 MB [file spectrum.00184-22-s0001.pdf]

Table S1 Patient's clinical data and characteristics of analyzed strains.

Species	Strains	Isolation Date	Age	Gender	Sample	Ward
P. aeruginosa	TL1671	11/04/2015	74	M	Wound	Endocrinology
	TL1736	20/05/2015	44	M	Sputum	Neurosurgery
	TL1744	23/05/2015	63	M	Sputum	ICU
	TL2314	08/03/2016	66	M	Sputum	ICU
	TL2917	09/02/2017	58	M	Sputum	ICU
	TL2967	13/03/2017	26	M	Sputum	Emergency
	TL3008	05/04/2017	67	M	Sputum	Neurosurgery
	TL3086	24/05/2017	65	M	Sputum	Neurosurgery
E. cloacae	CG648	26/05/2015	44	M	blood	Hematology
	CG737	12/09/2015	72	M	Wound	Endocrinology

	CG741	15/09/2016	72	M	tissue	Endocrinology
	CG884	24/04/2016	70	F	Drainage fluid	ICU
	CG1050	30/09/2016	81	F	blood	geriatric
	CG1051	04/10/2016	81	M	catheter	geriatric
	CG1479	08/01/2018	37	M	Urine	Urology
	CG1574	07/05/2018	51	F	ascites	Hematology
E. coli	DC90	27/03/2012	76	M	Wound	Gastrointestinal Surgery
	DC3737	05/05/2015	52	M	Wound	Orthopedics
	DC3806	19/05/2015	17	F	Sputum	Hematology
	DC3846	28/05/2015	77	F	Urine	Urology
	DC4887	23/02/2016	63	M	Urine	Urology
	DC5262	18/05/2016	32	F	Urine	Emergency
	DC5286	23/05/2016	82	F	Urine	Endocrinology

	DC7333	18/05/2016	32	M	Drainage fluid	ICU
	FK20	08/03/2012	88	F	Sputum	Neurology
	FK150	19/06/2012	60	M	Pus	Emergency
	FK169	02/07/2012	79	F	Sputum	General
K. pneumoniae	FK1913	19/01/2015	74	M	Sputum	Neurosurgery
	FK1986	03/03/2015	44	M	Blood	Endocrinology
	FK3810	17/03/2017	21	F	Stool	ICU
	FK6663	04/05/2019	73	M	Stool	ICU
	FK6696	09/05/2019	52	M	Bile	Emergency

Abbreviations: M, Male; F, Female.
